# Supplementary figures and images for: Computational mechanisms underlying cortical responses to the affordance properties of visual scenes
Source: PLoS Comput Biol. 2018 Apr 23;14(4):e1006111. doi: 10.1371/journal.pcbi.1006111 (PMC5933806; doi:10.1371/journal.pcbi.1006111)

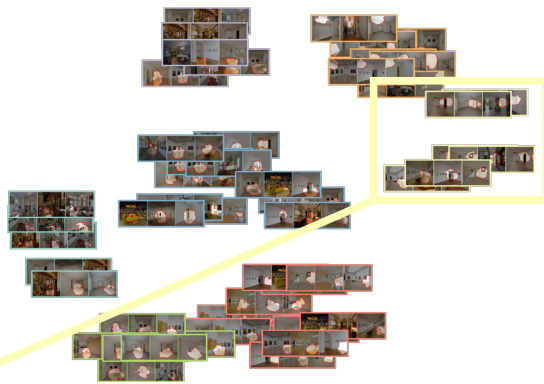

Cluster 2

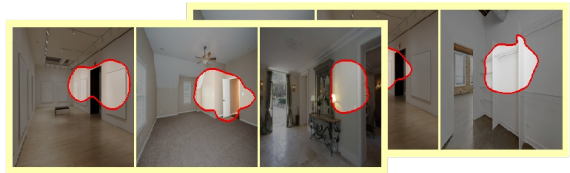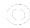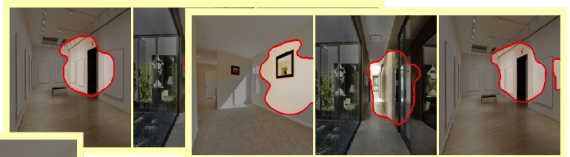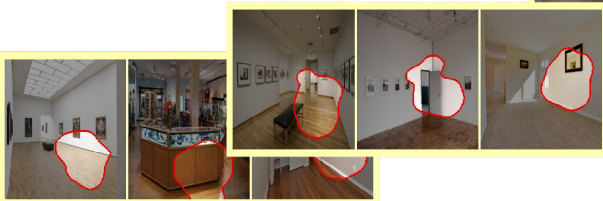

Supplement: S2 Fig — (PDF) [file pcbi.1006111.s002.pdf]

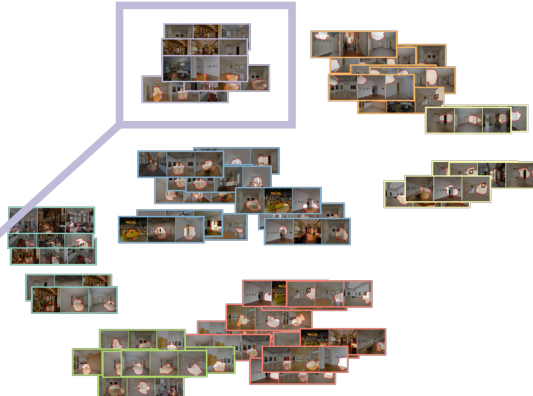

Cluster 3

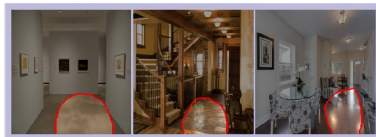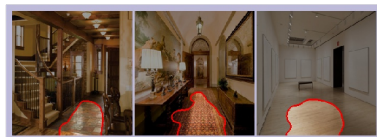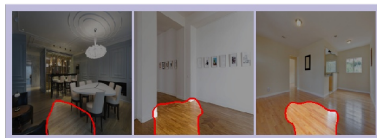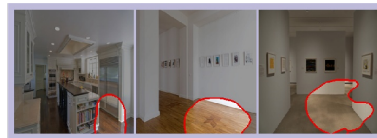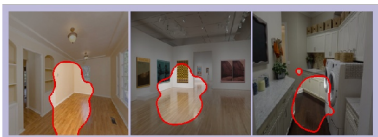

Supplement: S3 Fig — (PDF) [file pcbi.1006111.s003.pdf]

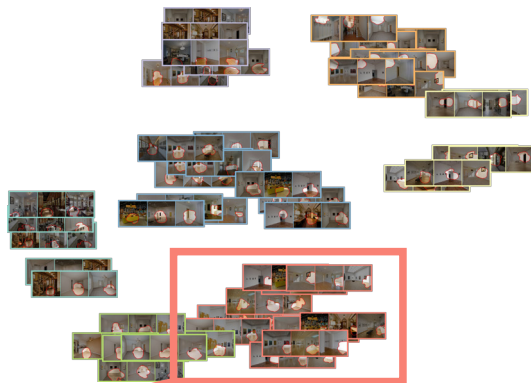

Cluster 4

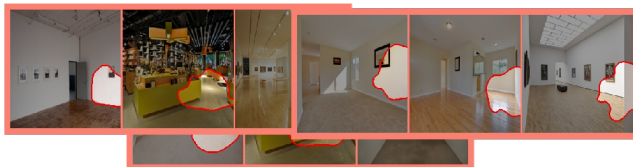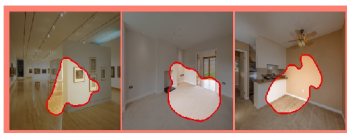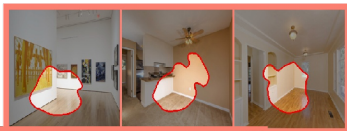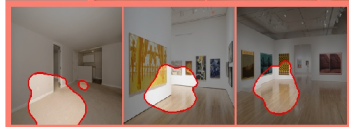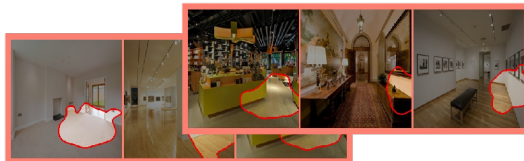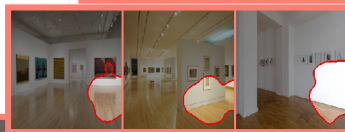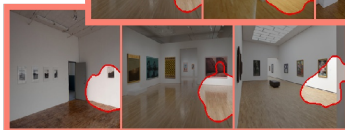

Supplement: S4 Fig — (PDF) [file pcbi.1006111.s004.pdf]

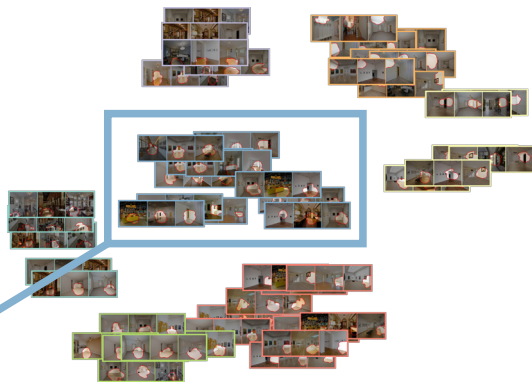

Cluster 5

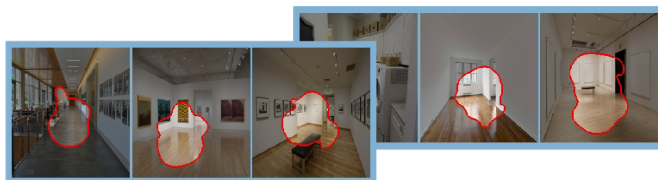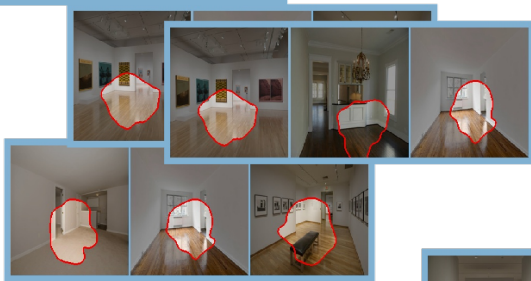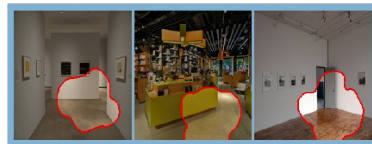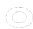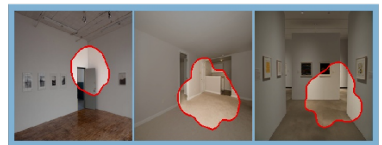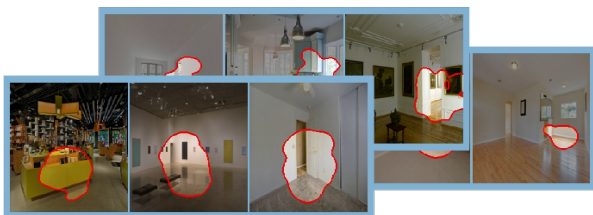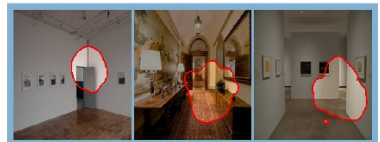

Supplement: S5 Fig — (PDF) [file pcbi.1006111.s005.pdf]

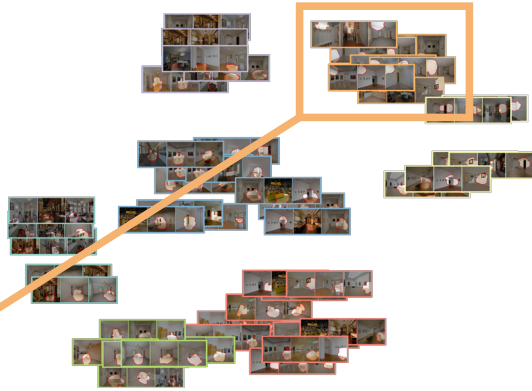

Cluster 6

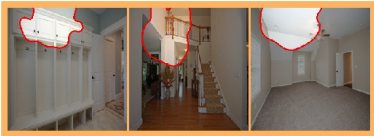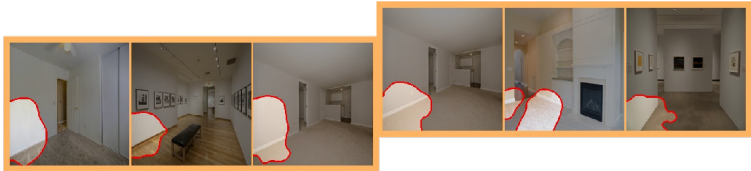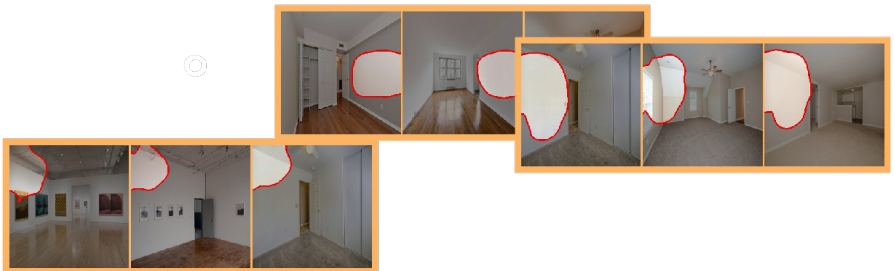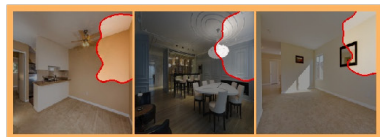

Supplement: S6 Fig — (PDF) [file pcbi.1006111.s006.pdf]

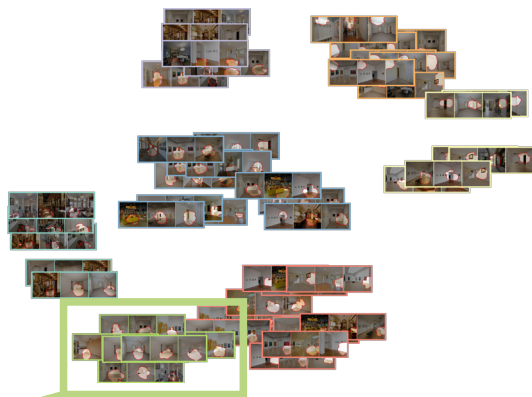

Cluster 7

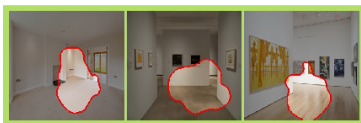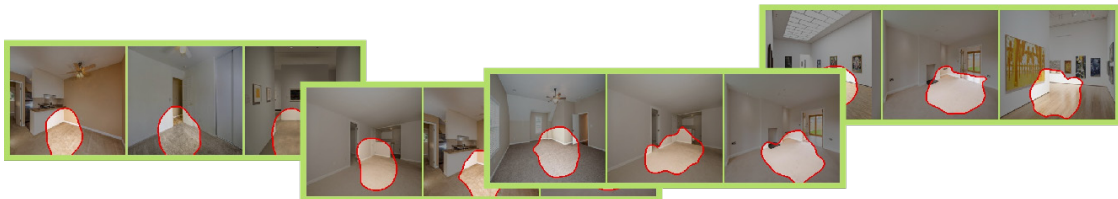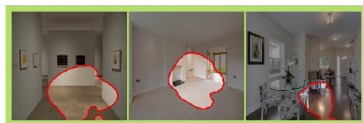

Supplement: S7 Fig — (PDF) [file pcbi.1006111.s007.pdf]

A

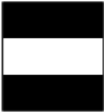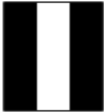

B

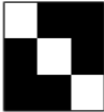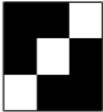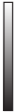

2

-1

Supplement: S8 Fig — Each kernel spans 3 pixels by 3 pixels. (A) Kernels used to extract lines at cardinal orientations (0 and 90 degrees). (B) Kernels used to extract lines at oblique orientations (45 and 135 degrees). (PDF) [file pcbi.1006111.s008.pdf]
