# Supplementary material for: Computational mechanisms underlying cortical responses to the affordance properties of visual scenes
Source: PLoS Comput Biol. 2018 Apr 23;14(4):e1006111. doi: 10.1371/journal.pcbi.1006111 (PMC5933806; doi:10.1371/journal.pcbi.1006111)
Supplement: S1 Fig — (PDF) [file pcbi.1006111.s001.pdf]

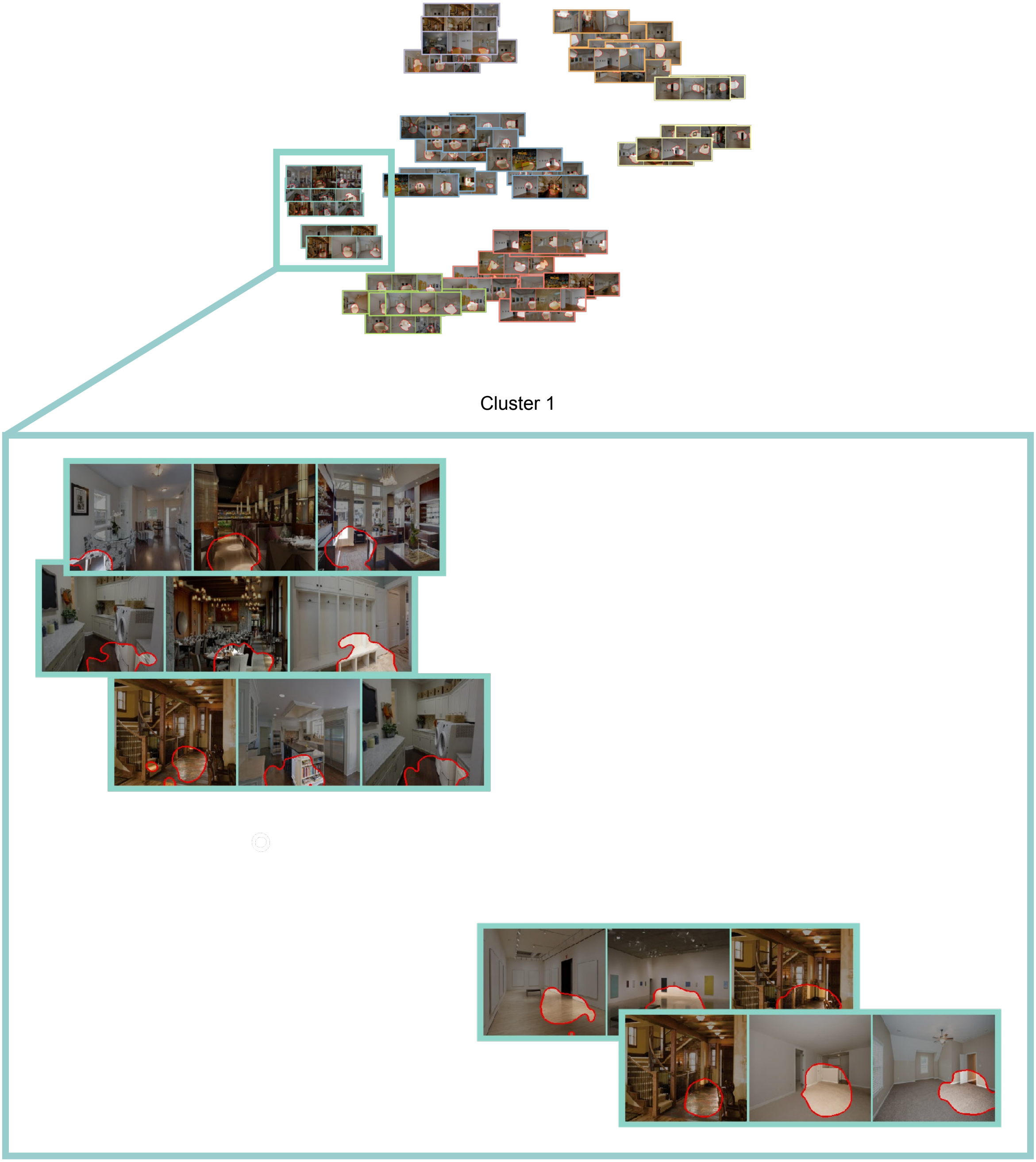
Cluster 1
